# Emerging *Echinococcus Shiquicus* Infection of Asian Badgers in the Qinghai–Tibetan Plateau

**DOI:** 10.1155/2023/6874033

**Published:** 2023-11-23

**Authors:** Yong Fu, Xueyong Zhang, Zhi Li, Zhenghe Shi, Xiao Ma, Ru Meng, Qing Zhang, Cunzhe Zhao, Shuai Guo, Wanli Ma, Hong Duo, Yuting Zhao, Faming Wu, Donglei Sun, Xiuying Shen, Yijuan Ma, Gongguan Liu, Zhihong Guo

**Affiliations:** ^1^Qinghai Academy of Animal Sciences and Veterinary Medicine, Qinghai University, Xining, China; ^2^Qinghai Provincial Key Laboratory of Pathogen Diagnosis for Animal Diseases and Green Technical Research for Prevention and Control, Xining, China; ^3^Qinghai Institute for Endemic Disease Prevention and Control, Xining, China; ^4^Xining Animal Disease Control Center, Xining, China; ^5^The Research Key Laboratory for Echinococcosis of Qinghai Province, Qinghai University, Xining, China; ^6^College of Agriculture and Husbandry, Qinghai University, Xining, China; ^7^Dangluo Town Animal Husbandry and Veterinary Station, Maqin, China; ^8^Department of Genetics and Developmental Biology, School of Life Sciences and Biotechnology, Shanghai Jiao Tong University, Shanghai, China; ^9^Key Laboratory of Animal Disease Diagnostics and Immunology, Ministry of Agriculture, MOE International Joint Collaborative Research Laboratory for Animal Health & Food Safety, College of Veterinary Medicine, Nanjing Agricultural University, Nanjing, China

## Abstract

Echinococcosis is a zoonotic disease currently causing significant public health issues worldwide. The emerging and the expansion of *Echinococcus* spp. tapeworms in wildlife species and habitats are indeed underrecognized. Here, using infrared camera surveillance followed by morphological and genetic characterization, *Echinococcus shiquicus* (*E. shiquicus*), tapeworms were unexpectedly detected from Asian badgers in the Qinghai–Tibetan Plateau of China for the first time. In specific, an area of 3,939 km^2^ at an altitude of 3,691–5,339 m above sea level was monitored, from which fecal samples were collected, and fecal DNA was sequenced to solidify its match with the genome of Asian badgers before fecal egg examination. We further revealed that the isolated fecal eggs were morphologically representing *E. shiquicus* being oval in shape and containing a hexacanth embryo, and genetically formed a unique clade with diverse registered *E. shiquicus* isolates as illustrated by phylogenetic analysis. Overall, our investigation suggested Asian badger as a potential new definitive host of *E. shiquicus* tapeworm. More extensive surveillance for *E. shiquicus* tapeworm should be conducted in neglected host species and their habitats in the Qinghai–Tibetan Plateau.

## 1. Introduction

Echinococcosis, caused by infection with the metacestode stage of *Echinococcus* species, is listed as one of the 17 neglected tropical diseases by WHO [1, 2]. Currently, there are at least nine recognized *Echinococcus* species, among which *Echinococcus shiquicus* (*E. shiquicus*) was first recorded as a new species from the Qinghai–Tibetan Plateau of China in 2005 [3]. *E. shiquicus* was originally thought to be transmitted only between the Tibetan fox (*Vulpes ferrilata*, as definitive hosts) and the plateau pika (*Ochotona curzoniae*, as intermediate host) [3]. Subsequently, various small mammals were found infected with *E. shiquicus* [4, 5], and dog was confirmed as the potential definitive host by copro-DNA PCR and sequencing [6, 7]. However, the zoonotic transmission potential of *E. shiquicus* has not been currently determined [8, 9]. Thus, it is urgent to unearth the potential host range and further elucidate the zoonotic potential (if any) of *E. shiquicus* within the Qinghai–Tibetan Plateau.

The Asian badger (*Meles leucurus*), as a widespread species, is distributed throughout the mainland of China [10]. The badger, an opportunistic forager of predation, has adapted to using the available trophic resources depending on environmental characteristics [11], particularly the abundant biomass of small mammals (such as plateau pikas and plateau voles) on the Qinghai–Tibetan Plateau as its main diet. This dietary habit might pose the badger at risk of infection by preying on these small mammals carrying pathogens.

We hereby showed that the eggs isolated from the fecal samples from the Asian badger were morphologically and genetically representing *E. shiquicus*. For the first time, we uncovered Asian badger as the potential definitive host of *E. shiquicus* by screening the neglected high altitudes across the Qinghai–Tibetan Plateau.

## 2. Materials and Methods

Our study was conducted in the southeastern part of the Qinghai–Tibetan Plateau (located in the Dangluo town of Maqin county), which covers an area of 3,939 km^2^ (99°10′–100°38′ E and 33°44′–34°36′ N), and an altitude of 3,691–5,339 m above sea level. The town is subjected to a plateau continental semi-humid climate and is located in the “Sanjiangyuan National Nature Reserve” in China. In September 2021, the study area was divided into grids of 0.01 km^2^ for systematic placement of infrared cameras, which traced the tracks of Asian badgers and were positioned at locations to maximize their photo captures.

**Figure 1:**
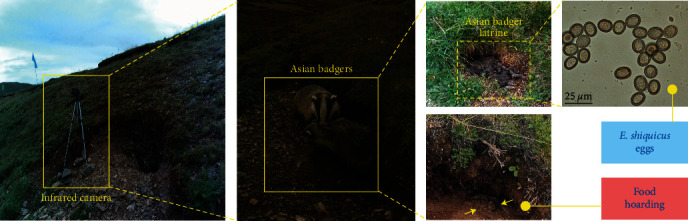
Infrared camera screening for Asian badgers and microscopic fecal egg examination. Infrared camera trap image of Asian badgers from the study area (far left and middle left), showing the defecating at latrines and food hoarding (dead plateau pikas, arrow) behavior around the dens (middle right), and microscopic observation of *Echinococcus shiquicus* tapeworm eggs (far right).

Interestingly, we found Asian badgers defecating at a fixed place and food hoarding (dead plateau pikas) behavior around the dens (Figure 1). The feces, which are strip-like and about 1–2 cm in thickness, are distributed in piles, which is consistent with the findings of a previous appearance study [12]. Next, the feces were collected from the various dunghills and were labeled and held separately in plastic bags in the field, then being stored at −80°C for at least 10 days to kill pathogenic eggs [13]. Fecal DNA and egg DNA were extracted using the TIANamp Stool DNA Kit (TIANGEN Biotech Co., Ltd., Beijing, China) according to the manufacturer's instructions. The host origin of feces was further determined by using a PCR targeting the D-loop region of mitochondrial DNA [14], based on the fecal morphological characteristics. Fecal egg examination was performed routinely on 1.0 g of feces by the centrifugal flotation method using 1.27 g/mL sucrose; when taeniid eggs were detected from the feces by microscopic examination, the eggs were collected for the molecular identification of their species [15]. Meanwhile, the partial mitochondrial *cox1* gene (444 bp), a molecular marker for *Echinococcus* species identification, was used to identify *Echinococcus* species as described previously [16].

## 3. Results and Discussion

**Figure 2:**
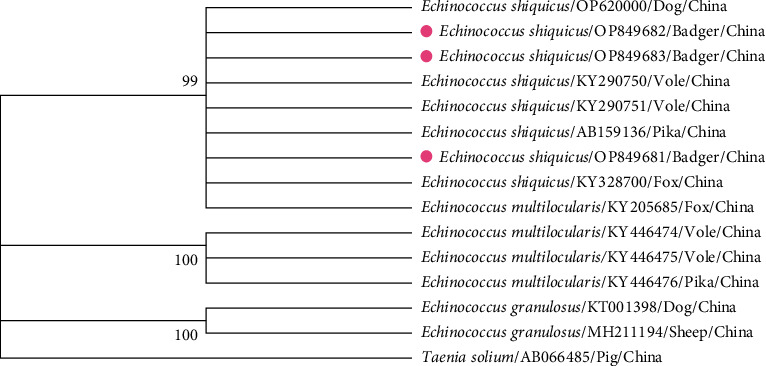
Phylogenetic analysis of fecal eggs from Asian badgers. Maximum likelihood analysis of the partial sequences of mitochondrial *cox1* gene inferred from isolates of *Echinococcus* spp., using the kimura 2-parameter model. Taenia solium was used as an outgroup. The phylogenetic tree was constructed with MEGA7.0, and the bootstrap values >50% are shown for the nodes that appeared along the branches with 1,000 replicates. Pink circle, the *cox1* gene amplified in this study.

We successfully determined nine fecal samples, and sequences were matched to published sequences of *M. leucurus* (GenBank accession no. LC333397.1). The results showed the eggs were oval in shape and contained hexacanth embryo, which is consistent with mature *E. shiquicus* eggs from a previous microscopic observation [3] (Figure 1). Importantly, BLAST (http://blast.ncbi.nlm.nih.gov) analysis of the tapeworm eggs amplicons (3/9, 33.3%) was identical to *E. shiquicus* (99.3%–100%) registered in GenBank, and the phylogenetic analysis showed the positive samples identified (OP849681–OP849683) formed a single well-supported clade with diverse isolates *E. shiquicus* registered in GenBank (Figure 2). Taken together, these findings suggested that the Asian badger is a potential definitive host of *E. shiquicus*.

**Figure 3:**
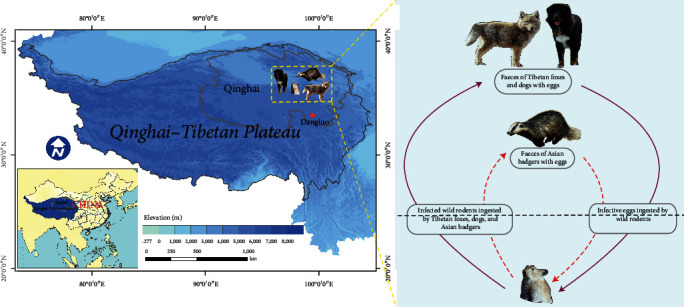
Proposed life cycle of *Echinococcus shiquicus* involving Asian badgers in the Qinghai–Tibetan Plateau, China. Study area location in the Qinghai–Tibetan Plateau (left) and the life cycle (right) of *E. shiquicus* involves intermediate hosts (such as plateau pikas and plateau voles) and definitive hosts, namely Tibetan foxes and dogs in family Canidae and herein identified Asian badgers in family Mustelidae, from the Qinghai–Tibetan Plateau.

Until now, *E. shiquicus* has only been found in the Qinghai–Tibetan Plateau of China. Some studies have demonstrated that it is an indigenous parasite, and this is probably due to the evolving within predator–prey cycles of endemic mammal species from the Qinghai–Tibetan Plateau [17]. Therefore, we speculated that *E. shiquicus* has complex wildlife–parasite cycles in the Qinghai–Tibetan Plateau. In this study, we found that the Asian badgers exhibit a distinct predatory preference for the plateau pikas in the Qinghai–Tibetan Plateau, and this specific food chain increased *E. shiquicus* infection opportunities for badgers (Figure 3). Altogether, these findings suggested that adaptive evolution over long periods of the ecological isolation promoted *E. shiquicus* adaptation to the local environment.

The Qinghai–Tibetan Plateau, with its unique climatic, topographical, and biological characteristics, is an isolated geographical area with important localities for the evolution of various organisms [4]. Not surprisingly, a variety of *Echinococcus* species coexist in the Qinghai–Tibetan Plateau area, and some emerging/re-emerging echinococcosis evolve to exploit some new hosts [5, 6]. Although the *E. shiquicus* life cycle is relatively fixed, it is perpetuated to a large extent by wildlife–parasite interactions to the transmission dynamics [18]. Our studies indicate that *E. shiquicus* infected Asian badgers in this area, and the potential role for Asian badgers in the transmission of *E. shiquicus* remains to be elucidated. In addition, there are presently no data available on the zoonotic potential of *E. shiquicus*, and it's worrisome that *E. shiquicus* may create significant public health issues similar to those of other *Echinococcus* species [19, 20], which contribute to human infections. In future studies, it is essential to go into the transmission mechanism of Asian badgers naturally infected with *E. shiquicus* for the prevention of this parasitic disease. Furthermore, there remains a dearth of data available on the epidemiological status of *E. shiquicus*, and more extensive surveillance is necessary to thoroughly understand the possibility of humans and other wildlife animals being infected by the transmission dynamics of *E. shiquicus*.

## Data Availability

The data that support the findings of this study are available from the corresponding author upon reasonable request.
